# Resveratrol and Dulaglutide ameliorate adiposity and liver dysfunction in rats with diet-induced metabolic syndrome: Role of SIRT-1 / adipokines / PPARγ and IGF-1

**DOI:** 10.1007/s40199-023-00458-y

**Published:** 2023-03-29

**Authors:** Hanan Abdel Moneam A. Shamardl, Noha A. Ibrahim, Dina H. Merzeban, Azza M. Elamir, Rehab M. Golam, Asmaa M. Elsayed

**Affiliations:** 1 Medical Pharmacology Department, Faculty of Medicine, Fayoum University, Fayoum, 19052 Egypt; 2 Histology and Cell Biology Department, Faculty of Medicine, Fayoum University, Fayoum, 19052 Egypt; 3 Medical Physiology DepartmentFaculty of Medicine, Fayoum University, Fayoum, 19052 Egypt; 4 Medical Biochemistry and Molecular Biology Department, Faculty of Medicine, Fayoum University, Fayoum, 19052 Egypt

**Keywords:** Dulaglutide, Resveratrol, Metabolic syndrome, SIRT-1, Adipokines, IGF-1

## Abstract

**Background:**

Adiposity and non-alcoholic fatty liver disease (NAFLD) are common characteristics of metabolic syndrome (MS). Understanding the underlying pathogenesis is crucial for the development of new remedies. Resveratrol controls obesity and glycemic disorders in patients with MS.

**Objectives:**

This study aimed to evaluate the effect of resveratrol and dulaglutide on adipose tissues and liver in rats with MS, declaring their possible mechanisms.

**Methods:**

Rats allocated as Control, MS (induced by a high fat/ high sucrose diet for eight weeks), MS + Resveratrol (30 mg/kg/day orally), and MS + Dulaglutide (0.6 mg/kg twice weekly SC); drugs administration was in the last four weeks. Serum biochemical measurements were done. Liver and visceral fat were processed for biochemistry, histopathology, and immunohistochemistry.

**Results:**

MS results demonstrated significantly increased systolic and diastolic blood pressure, anthropometric measurements, serum levels of alanine aminotransferase (ALT), glycemic indices, and lipids with decreased HDL-C. Tissue levels of leptin, malondialdehyde (MDA), and TNF-α reactivity significantly increased. Expression of adiponectin, PPARγ, and insulin growth factor-1 (IGF-1) decreased. Also, Western blotting mRNA gene expression of liver SIRT-1 was down-regulated. Resveratrol and dulaglutide significantly and effectively reversed MS complexity, ameliorating all findings, particularly NAFLD and adiposity-induced inflammation. Resveratrol significantly appears superior to dulaglutide regarding the effects on hemodynamics, lipids, adipokines, IGF-1 levels, and adipocyte size. Parallel, dulaglutide has more influence on glycemic control.

**Conclusion:**

Protective effects of the drugs may be through correlations between SIRT-1/adipokines/IGF-1 and PPARγ, improving the cross-talk between insulin resistance, obesity markers, liver dysfunction, and TNF-α. Promising multi-beneficial therapies of resveratrol or dulaglutide in MS are recommended clinically for this purpose.

**Graphical Abstract:**

Showing the Experimental Design

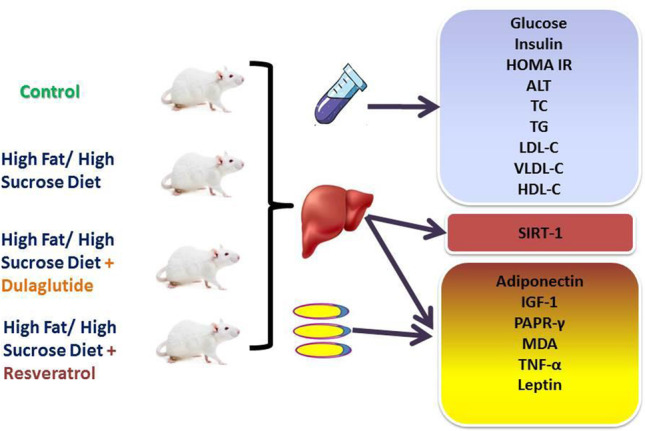

## Introduction

Metabolic syndrome (MS) is an interconnected combination of biochemical, clinical, and metabolic factors. MS manifests as adipose tissue expansion, insulin resistance (IR), type 2 diabetes mellitus (T2DM), elevated blood pressure, and cardiovascular disease. The prevalence of MS ranges from < 10% to more than 84% worldwide, depending on the geographic distribution, the guidelines for diagnosis used, and differences in the studied population regarding their race, age, sex, and ethnicity [1, 2]. The MS prevalence frequently coincides with obesity and T2DM prevalence. According to the National Health and Nutrition Examination Survey (NHANES), the MS prevalence represents 60% among obese people, 22% among overweight, and 5% among normal weights [3]. Moreover, about 70–80% of diabetic patients have been diagnosed with MS [4].

Therefore, increasing lipolysis in adipose tissues surges hepatic free fatty acid uptake, lipid accumulation, and fatty liver disease [5]. Adipose tissue remodeling associated with obesity alters the synthesis of adipokines such as tumor necrosis factor (TNF-α), leptin, and adiponectin. Leptin and TNF-α are positively correlated with body obesity and contribute to promoting inflammation and IR development [6]. Adiponectin may be the only adipokine decreases in obesity and MS. It is also negatively correlated to hepatic IR and liver fat content [7].

Insulin-like growth factor-1 (IGF-1) is a hepatokine expressed locally in the liver in addition to many other tissues, particularly adipose tissues. Low IGF-1 levels are observed in obesity-induced liver dysfunction [8, 9]. So, IGF-1, leptin, and adiponectin are considered non-invasive biomarkers for non-alcoholic fatty liver diseases (NAFLD) diagnosis [10].

Sirtuin -1 (SIRT-1) is a nicotinamide adenine dinucleotide (NAD +)—dependent deacetylase. It acts as a fuel-sensing molecule that regulates ATP synthesis and helps to restore the body's energy balance. SIRT-1 may be crucial in lowering fat deposition and improving glucose tolerance and insulin sensitivity, which may therefore improve all of the symptoms of MS, especially adiposity and fatty liver [11].

Resveratrol is a natural polyphenol compound and SIRT-1 activator. It has many biological and pharmacological properties, including anti-inflammatory and antioxidant effects, suggesting a role in MS treatment and its related disorders [12]. Resveratrol supplementation improved glucose tolerance by modulating the glucose metabolism in skeletal muscle and liver in pigs with MS [13]. It also positively influenced the hepatic oxidative stress (OS) in a rat model of MS with a high fructose diet [14]. The anti-obesity effect of resveratrol is through decreasing lipogenesis and increasing lipolysis in adipose tissues [15]. Moreover, resveratrol increases the brown adipose tissue activity to restore thermogenesis [16].

Dulaglutide, a glucagon-like peptide-1(GLP-1) receptor agonist, is approved for T2DM and obesity treatment. It mediates insulin release in response to elevated glucose levels, increases insulin sensitivity, decreases glucagon secretion, modifies β-cell damage, delays stomach emptying, and reduces appetite [17]. Several studies reported that GLP-1 agonist therapy is effective in the reduction of body weight and NAFLD [18, 19].

Since obesity and NAFLD are strongly associated with T2DM and IR in MS, and their current management with weight loss and exercise is insufficient, the discovery of novel therapeutic alternatives has become necessary. Dulaglutide is efficient and well-tolerated**,** although it could still have different adverse effects, such as gastrointestinal symptoms, low risk of acute pancreatitis, and renal damage [17]. Therefore, the current focus is introducing a safe medicine (or more) of natural origin that is used for long periods without experiencing many side effects, like resveratrol. So, we conducted this study to evaluate the effectiveness of resveratrol versus dulaglutide on the adipose tissue and liver in a metabolic syndrome rat model, investigating the possible underlying mechanisms.

### Materials and methods

#### Animals

Twenty-four male Wistar rats aged one month, weighing 130–150 g were maintained under conventional laboratory conditions of temperature (20 ± 5ºC) with a regular 12 h light/dark cycle throughout the study.

#### Ethical statement

The Ethics Committee of Research, Faculty of Medicine, Fayoum University approved the experimental protocol of this study (R226 / 92–2022). All procedures of the experiment followed the standard guidelines and regulations of the US National Research Council "Guide for the Care and Use of Laboratory Animals.”

#### Experimental design

Rats were divided into four groups (six rats/each): Normal Control group fed ad libitum standard chow and tap water. Untreated metabolic syndrome (MS) group; fed a high-fat/high-sucrose (HF/HS) diet (5% sheep fat; 5 g fat /100 g of standard chow and 10% sucrose in drinking water for eight weeks). MS + Dulaglutide group (dulaglutide 0.6 mg/kg twice weekly injected subcutaneously, purchased from Eli Lilly, Japan) (corresponding 8 fold human equivalent dose (HED), based on plasma area under the curve [20] and MS + Resveratrol group (resveratrol was given 30 mg/kg daily orally, purchased from Xian Lukee Bio-Tech Co., Ltd.) [21]. Induction of metabolic syndrome in the last three groups was confirmed by elevated systolic and diastolic blood pressure, blood glucose > 200 mg/dl, and body mass index (BMI) > 0.7 g/cm^2^.

#### Anthropometric measurements

On the day of dissection, body weight (BW), body length, white visceral adipose tissues including mesenteric, perirenal, and epididymal fat were weighed, calculation of BMI (g/cm^2^) as Weight g / Length cm^2^, and Lee index [LI = body weight (g) ^1/3^ × 1000 / body length (cm)] [22].

#### Blood pressure measurement

A computerized non-invasive blood pressure system, CODA™ (Kent Scientific, Torrington, CT, USA) used to measure tail rat blood pressure weekly using volume pressure method. Values of systolic (SBP), diastolic (DBP) blood pressure, and heart rate (HR) were recorded.

#### Blood sampling and biochemical measurements

At the end of the experiment, all animals fasted for 12 h and underwent light anesthesia with inhaled diethyl ether. We collected intra-cardiac blood sample, centrifuged, and separated serum for determination of glucose, total cholesterol (TC), triglyceride (TG), high-density lipoprotein cholesterol (HDL-C), and alanine aminotransferase (ALT), kits produced by BioSystem S.A Costa Brava, Barcelona, Spain. Serum VLDL-C and LDL-C levels were calculated by using the Friedewald formula [23] as follows:

$$\begin{array}{l}\mathrm{VLDL}-\mathrm{C }=\mathrm{ TG}/5\\ \mathrm{LDL}-\mathrm{C }=\mathrm{ TC }-\mathrm{ HDL}-\mathrm{C}-\mathrm{ TG}/5.\end{array}$$

We measured serum insulin with ELISA kits (Cusabio, Inc., Houston, Texas, USA) according to the manufacturer’s instructions. Estimation of Homeostasis model assessment of insulin resistance (HOMA-IR) was by using the following formula [24]: HOMA-IR = fasting glucose (mg/dl) × fasting insulin (µU/ml) / 405.

#### Tissue sampling and homogenate preparation

After sacrification, we preserved specimens of the liver and visceral fat in 10% buffer-neutral formalin for histology and immunohistology study. Other parts were homogenized in 0.1-M phosphate buffer (pH 7.4) to get 20% w/v tissue homogenates which were centrifuged at 3000 × g for 30 min in 4 °C. The supernatants were collected, preserved at – 70 °C for biochemical measurements.

Lipid membrane damage was determined by measuring malondialdehyde (MDA) level used the Biodiagnostic kit [25]. We measured adiponectin and leptin levels using ELISA kits, according to the manufacturer’s instructions (Calbiotech, Austin, USA).

#### Quantitative assessment of Peroxisome Proliferator-Activated Receptor gamma (PPARγ) gene expression by real-time PCR

Total RNA was extracted from liver and adipose tissue homogenate utilizing Qiagen extraction kit (Qiagen, Valencia, CA, USA) following the manufacturer’s instructions. RNA concentrations and purity were measured with a Nanodrop ND-2000 spectrophotometer (Thermo Scientific Inc., USA) and kept at -80 °C. Reverse transcription of RNA was carried out using QuantiTect reverse transcription kit (Qiagen) as described in the manufacturer’s protocol. The expression of PPARγ gene was done by RT-thermal cycler (MJ Research Inc, Watertown, Massachusetts, USA) with a Fast Start DNA Master SYBR Green I kit (Roche Diagnostics, Indianapolis, IN, USA) following the manufacturer’s protocol. Glyceraldehyde-3 phosphate dehydrogenase (GAPDH) was utilized as an internal control for data normalization.

Real-time PCR was implemented in a total volume of 20 µl comprising: 2 µl of cDNA, 10 µl of Syber Green PCR Master Mix (Roche Diagnostics USA), and 10 pmol of each primer. Thermal cycling conditions were utilized, involving a 95ºC step for 10 min, followed by 40 cycles of 95ºC for15 seconds, 60ºC for 1 min, and 72ºC for 1 min. Relative expression of the intended gene mRNA was calculated utilizing the 2^−∆Ct^ method.

Primer sequences utilized for:

- PPARγ were; Forward 5ˋ- GCTACCGTTCCTCTATCAATGACA-3ˋ,
- Reverse 5ˋ- CAGATTTATTCAGCTTTGCCTCAG -3ˋ.
- GAPDH were; Forward 5ˋ- GTGACTTTATGGAGCCTAAGTTTG -3ˋ,
- Reverse 5ˋ- AGCTATAAATATGGCCAAGTCACT-3ˋ.

#### Western blotting of hepatic SIRT-1

Protein was extracted from liver tissues with RIPA lysis buffer supplemented with 1 mM phenylmethanesulfonyl fluoride and 1 mM protein phosphatase inhibitor. Then, the sample was centrifuged for 10 min at 12,000 r/min at 4 °C. The proteins were separated on 10% (w/v) SDS–polyacrylamide gels and transferred onto polyvinylidene fluoride membranes. The membranes were blocked with 5% (w/v) milk (Bio-Rad) for 2 h at room temperature and then incubated overnight at 4 °C with primary antibodies against SIRT-1 (Santa Cruz Biotechnology kit, Inc., Santa Cruz, California, USA) and β-actin (1:1000, Proteintech). Next, the membranes were washed with TBS with 0.05% Tween 20 (TBST) three times and incubated with horseradish peroxidase-conjugated secondary antibodies for 60 min. The signals were detected with a ChemiDocXRS + Imaging System (Tanon, Shanghai, China) [26].

#### Histopathology

The epididymal fat pads and liver specimens in 10% formalin were embedded in paraffin wax by routine protocol. 5 μm thick sections were stained with Haematoxylin and Eosin (H&E) for histological examination. Periodic acid–Schiff (PAS) histochemistry technique was used to demonstrate glycogen accumulation [27].

Immunohistochemical staining was done using: l. IGF-1 rabbit polyclonal antibody (Novus Biological USA, Catalog NO. NBP2-16929). Human breast carcinoma was used as a positive control. II. TNF-α rabbit polyclonal antibody (Thermo Fisher US, Catalog NO. PA5-120124), human lung carcinoma was the positive control (as provided by the manufacturer).The reaction of both IGF-1 and TNF-α was cytoplasmic. Negative control slides were done by omitting the primary antibodies.

#### Quantitative morphometric study

The following parameters were measured by using “Toup view” image analyzer computer system (China): The diameters of 20 randomly selected adipocytes from H&E-stained sections of the epididymal fat at × 100 magnification [28]. Area percent of IGF-1 and TNF-α immune-stain in adipose and liver tissues, area percent of hepatic PAS reaction were taken in ten randomly selected non-overlapping fields/slide at × 100 magnification.

#### Statistical analysis

We used SPSS, version 18.0, for data analysis after being checked for completeness and normality. We expressed it as mean and standard deviation (SD), using One-way ANOVA (analysis of variance) for comparisons of study variables between groups, followed by post-hoc (Tukey test) for multiple inter-group comparisons. Pearson correlation was used to test the association between quantitative study variables. We considered the data significant at *p* value < 0.05.

### Results

#### Effects of treatment on the anthropometric measurements

**Fig. 1:**
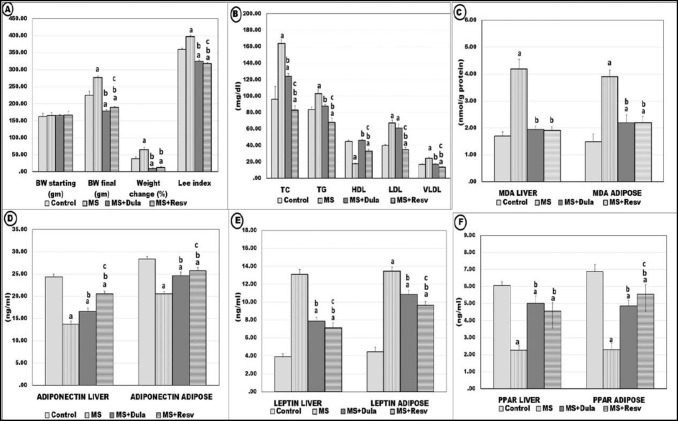
Effect of dulaglutide and resveratrol on different parameters in all experimental groups; (**A**): Starting body weight (BW starting), final body weight (BW final), weight change percent, and Lee index, (**B**): Serum total cholesterol (TC), triglyceride (TG), high-density lipoprotein-C (HDL-C), low-density lipoprotein-C (LDL-C) and very low-density lipoprotein-C (VLDL-C), **(C):** Malondialdehyde (MDA), (**D**): Adiponectin, (**E**): Leptin, (**F**): PPARγ gene expression. Abbreviations: MS: Untreated metabolic syndrome group, MS + Dulaglutide: dulaglutide treated group. MS + Resveratrol: Resveratrol treated group, Data represent mean ± SD, using One-way ANOVA test for comparisons of study variables between groups, followed by post-hoc Tukey test for multiple inter-group comparisons. Significance difference at *P*-value < 0.05. ^**a**^ Significant compared to control, ^**b**^ Significant compared to MS group, ^**c**^ Significant compared to MS + Dulaglutide group

Compared to the control group, the untreated MS group showed positive indicators of obesity: including a significant increase in BW, body gain percentage, BMI, LI, and visceral fat weight (*p < *0.05). While treatment with dulaglutide and resveratrol showed significantly decreased values of all these parameters compared to MS (*p < *0.05). With non-significant changes between the results of the two drugs (Table 1, Fig. 1A).

**Table 1:** Effects of treatment on anthropometric, hemodynamic, biochemical, and histologic parameters in experimental groups

	Control	MS	MS + Dulaglutide	MS + Resveratrol
SBP (mmHg)	114.88 ± 3.94	159.38 ± 4.81^a^	132.50 ± 3.82^a,b^	121.38 ± 3.85^a,b,c^
DBP (mmHg)	82.75 ± 2.60	108.00 ± 2.14^a^	93.88 ± 3.56^a,b^	86.38 ± 6.07^b,c^
Heart rate (beat/min)	421.33 ± 3.98	354.17 ± 3.37^a^	390.83 ± 3.82 ^a,b^	385 ± 4.93^a,b^
Length (cm)	16.80 ± .42	16.41 ± 0.38	17.74 ± 0.68^b^	18.29 ± 0.56^b^
BMI (g/cm^2^)	0.77 ± 0.03	1.01 ± 0.09	0.59 ± 0.06^a,b^	0.59 ± 0.02^a,b^
Mesenteric fat (gm)	2.12 ± 0.30	4.65 ± o.3^a^	2.11 ± 0.18^b^	2.26 ± 0.42^b^
Peri-renal fat (gm)	2.58 ± 0.32	5.08 ± 0.21^a^	2.96 ± 0.10^a,b^	2.85 ± 0.29^b^
Epididymal fat (gm)	3.02 ± 0.26	4.90 ± 0.37^a^	2.90 ± 0.13^b^	2.94 ± 0.32^b^
Total fat (gm)	8.27 ± 0.64	14.26 ± 1.18^a^	7.68 ± 0.28^b^	7.91 ± 1.36^b^
FBG (mg/dl)	70.88 ± 3.60	297.75 ± 7.21^a^	96.00 ± 4.21^a,b^	118.00 ± 1.69^a,b,c^
Insulin (µU/ml)	3.16 ± .26	5.29 ± 1.03^a^	3.07 ± .39^b^	3.06 ± 0.10^b^
HOMA-IR	0.56 ± 0.02	3.74 ± 0.07^a^	0.79 ± 0.01^a,b^	0.87 ± 0.03^a,b,c^
ALT (U/L)	38.00 ± 0.89	52.00 ± 0.89^a^	43.17 ± 1.47^a,b^	42.00 ± 0.63^a,b^
Adipocytes diameter	30.53 ± 1.37	130.24 ± 3.47^a^	101.49 ± 2.39^a,b^	90.4 ± 1.73^a,b,c^
Liver PAS area%	31.85 ± 2.17	9.43 ± 0.68^a^	18.16 ± 5.5^a,b^	18.58 ± 1.7^a,b^

*ALT* alanine transaminase enzyme, *BMI* body mass index, *DBP* diastolic blood pressure, *FBG* fasting blood glucose, *HOMA-IR* homeostasis model assessment of insulin resistance, *MS* Untreated metabolic syndrome group, *PAS* Periodic acid–Schiff and *SBP* systolic blood pressure

Data represent mean ± SD, using One-way ANOVA for comparisons of variables between groups, followed by post-hoc Tukey test for multiple inter-group comparisons. Significance difference at *P*-value < 0.05

^a^ Significant compared to control

^b^ Significant compared to MS group

^c^ Significant compared to MS + Dulaglutide group

#### Effects of treatment on SBP, DBP, and HR

MS results showed significantly (*p* < 0.05) increased SBP, DBP, and decreased HR values in comparison to the control group. After treatment with dulaglutide and resveratrol, the levels of SBP and DBP decreased while HR increased compared with MS group. The reduction of SBP and DBP with resveratrol treatment was significant compared to dulaglutide (*p* < 0.05) (Table 1).

#### Effects of treatment on serum biochemical measurements

The mean values of serum glucose, insulin, and HOMA-IR in the MS group significantly increased compared to the control group (*p* < 0.05). The results of the two drugs showed a significant decrease compared to MS. Dulaglutide treatment decreased the values of serum glucose and HOMA-IR more than the resveratrol one (*p* < 0.05) (Table 1).

MS group revealed significantly increased serum TC, LDL-C, VLDL-C, TG, and ALT levels associated with decreased HDL-C compared with the control group (*p<*0.05). Dulaglutide and resveratrol-treated groups significantly reversed these parameters. In comparing the results of both drugs, resveratrol treatment significantly (*p* < 0.05) reduced TC, TG, LDL-C, and VLDL-C. While dulaglutide significantly increased HDL-C, with no significant changes between them on ALT level (*p* > 0.05) (Fig. 1B).

### Effects of treatment on the hepatic and adipose tissue measurements

#### Malondialdehyde, adiponectin, and leptin levels

The MS results showed significantly increased mean levels of malondialdehyde and leptin with decreased adiponectin (*p < *0.05). Compared to the MS group, dulaglutide and resveratrol treatment significantly reversed these levels (*p* ˂ 0.05) (Fig. 1C, D, E).

#### PPARγ gene expression

The Expression of hepatic and adipose PPARγ in the MS group was significantly decreased compared to the control group (*p* ˂ 0.05). Both drugs significantly increased the expression in comparison to the MS group. Meanwhile, there was a significant increase in adipose PPARγ with resveratrol treatment in comparison to dulaglutide (*p* ˂ 0.05) (Fig. 1F).

#### Western blotting of hepatic SIRT-1 protein

**Fig. 2:**
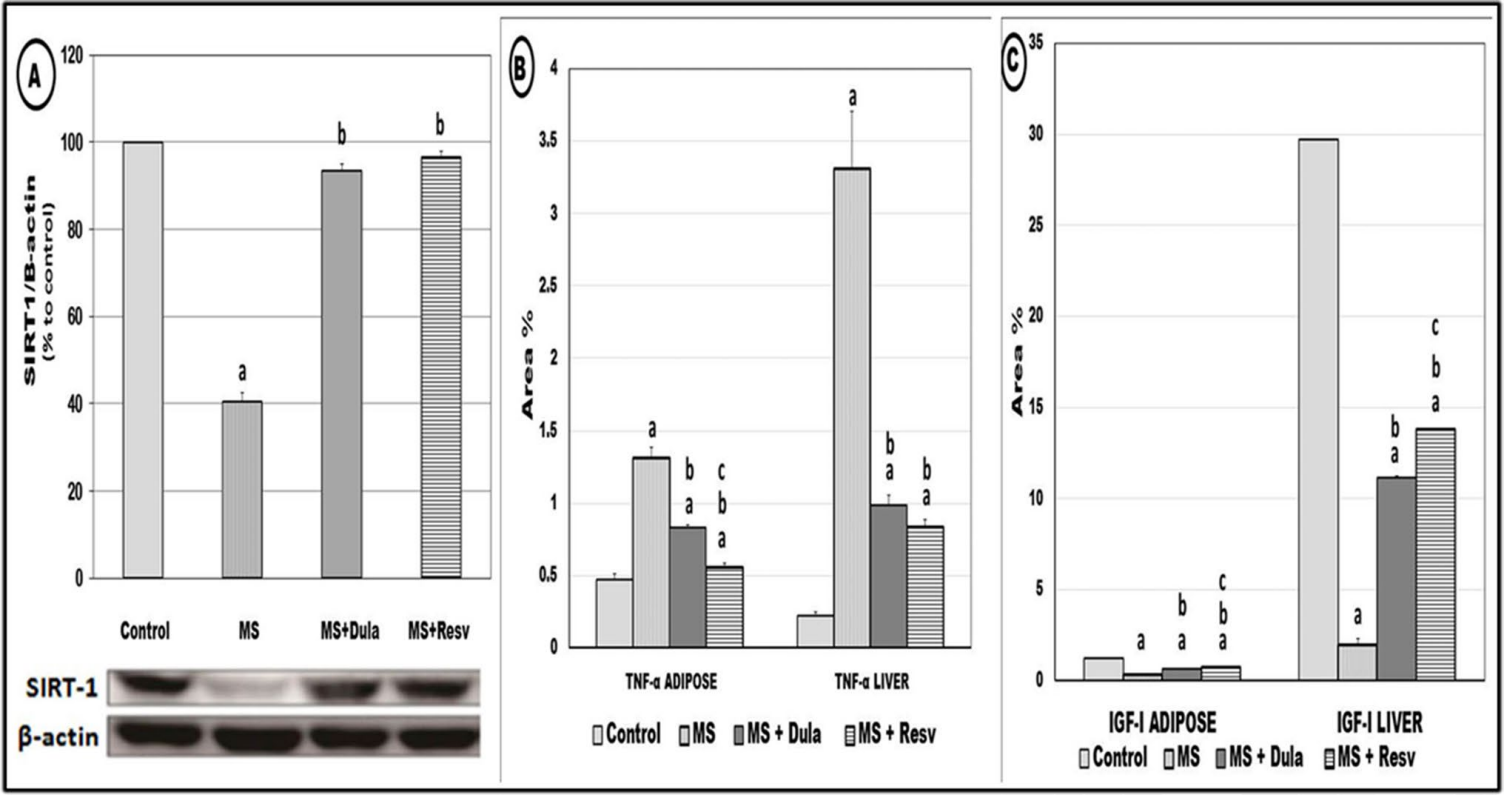
Effect of dulaglutide and resveratrol treatment on (**A**): Hepatic SIRT-1 protein expression, (**B**): Area percentage of Insulin growth factor-I (IGF-1) immuno-stain, (**C**):.Area percentage of Tumor necrosis factor- α (TNF- α) immuno-stain. Abbreviations: MS: Untreated metabolic syndrome group, MS + Dulaglutide: dulaglutide treated group. MS + Resveratrol: Resveratrol treated group, Data represent mean ± SD, using One-way ANOVA test for comparisons of study variables between groups, followed by post-hoc Tukey test for multiple inter-group comparisons. Significance difference at *P*-value ˂ 0.05. ^**a**^ Significant compared to control, ^**b**^ Significant compared to MS group, ^**c**^ Significant compared to MS + Dulaglutide group

The hepatic SIRT-1protein was downregulated in the MS group relative to the control group. While in dulaglutide and resveratrol groups, the expression showed a significant increase (*p* ˂ 0.05). There was no statistically significant difference between both drugs in the expression values (Fig. 2A).

#### Correlations analysis between different parameters

In the present work, oxidative and inflammatory biomarkers such as MDA and TNF-α were positively correlated, and with tissue leptin, while negatively associated with adiponectin, PPARγ, IGF-1, and SIRT-1. Adiponectin and PPARγ were positively correlated, and with tissues IGF-1 and SIRT-1, but negatively correlated with leptin. However, results of HOMA-IR were positively correlated with leptin and TNF-α, while negatively associated with adiponectin, PPARγ, IGF-1, and hepatic SIRT-1 (Table 2).

**Table 2:** Statistical correlations using Pearson Correlation test among different parameters

	Hepatic							
	Adiponectin	Leptin	PPARγ	TNF-α	IGF-I	MDA	PAS	SIRT-I
Adiponectin								
Leptin	−0.918*							
PPARγ	0.832*	−0.936*						
TNF-α	−0.851*	0.967*	−0.948*					
IGF-I	0.942*	−0.939*	0.873*	−0.856*				
MDA	−0.768*	0.903*	−0.895*	0.963*	−0.752*			
PAS	0.924*	−0.957*	0.907*	0.894*	0.991*	−0.799*		
SIRT-I	0.801*	−0.936*	0.926*	−0.991*	0.794*	−0.977*	0.837*	
HOMA-IR	−0.763*	0.915*	−0.918*	0.981*	−0.76*	0.985*	−0.809*	−0.996*
	Adipose							
	Adiponectin	Leptin	PPARγ	TNF-α	IGF-I	MDA	SIRT-I	
Adiponectin							0.912*	
Leptin	−0.918*						−0.767*	
PPARγ	0.945*	−0.891*					0.927*	
TNF-α	−0.948*	0.86*	−0.957*				−0.948*	
IGF-I	0.932*	−0.975*	0.903*	−0.888*			0.807*	
MDA	−0.915*	0.835*	−0.925*	0.951*	−0.867*		−0.945*	
HOMA-IR	−0.885*	0.729*	−0.903*	0.925*	−0.769*	0.931*	−0.996*	

*PPARγ* Peroxisome Proliferator-Activated Receptor gamma, *TNF-α* tumor necrosis factor alpha, *IGF-1* insulin growth factor 1, *MDA* malondialdehyde, *HOMA-IR* homeostasis model assessment of insulin resistance, *PAS* Periodic acid–Schiff

Data represent mean ± SD using One-way ANOVA test for comparisons of variables between groups. Pearson correlation was used to test the association between quantitative study variables. * Significance at *P*-value ˂ 0.05

### Histological results

#### Control group

**Fig. 3:**
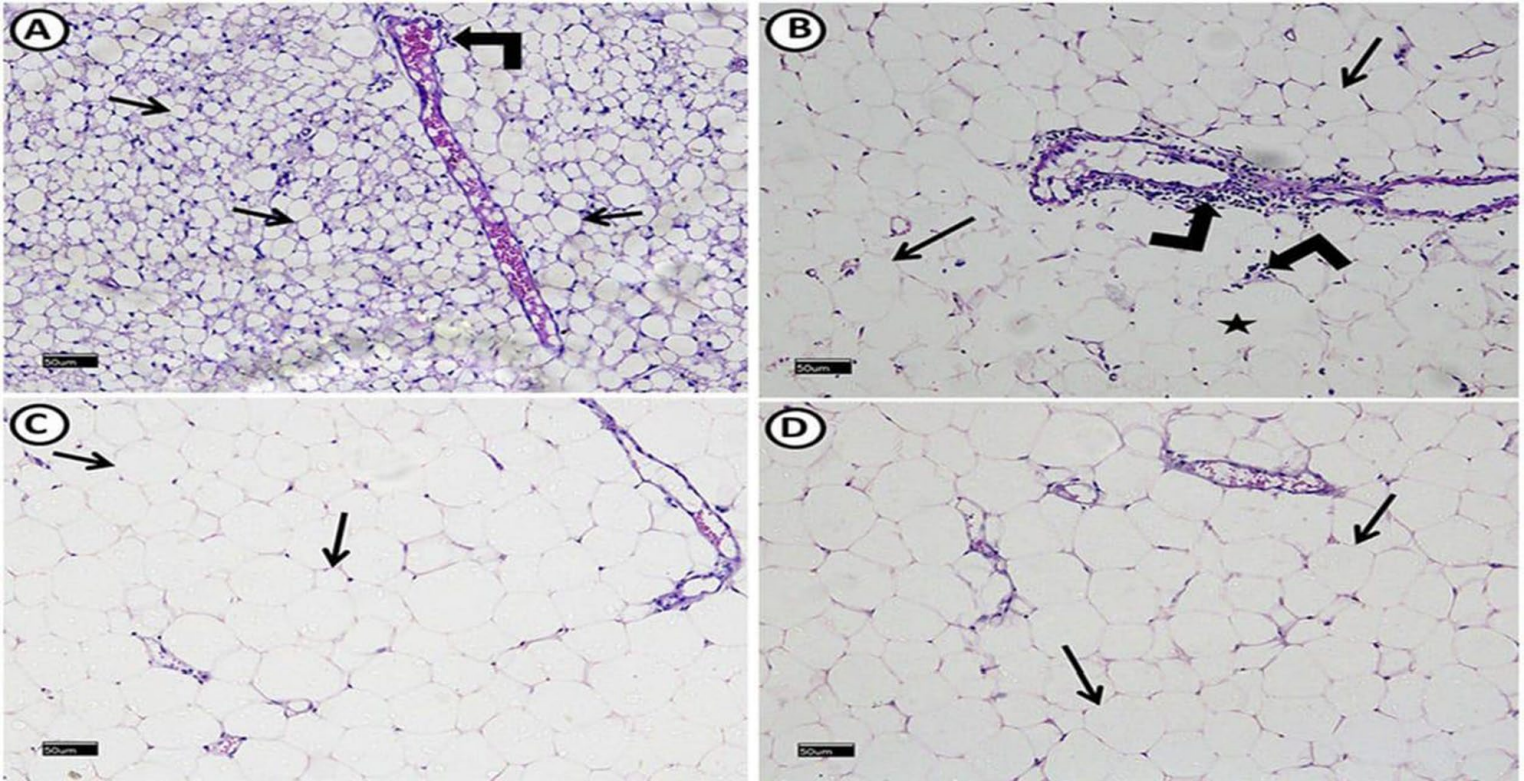
A photomicrograph of H&E stained epididymal white adipose connective tissue sections from all experimental groups: control group (**A**), reveals polyhedral unilocular adipocytes with peripheral nuclei and well-defined cell boundaries (arrows). Minimal perivascular inflammatory cells infiltration (right-angled arrows) can be detected. MS group, (**B**) shows apparently large adipocytes (arrows). Some adipocytes appear with ruptured cell boundaries (star). Evident perivascular inflammatory cells infiltration (right-angled arrows) also can be detected, MS + Dulaglutide (**C**) and MS + Resveratrol (**D**) groups show well-defined apparent large adipocytes (arrows). (H&E stain, Scale bar = 50 µm)

**Fig. 4:**
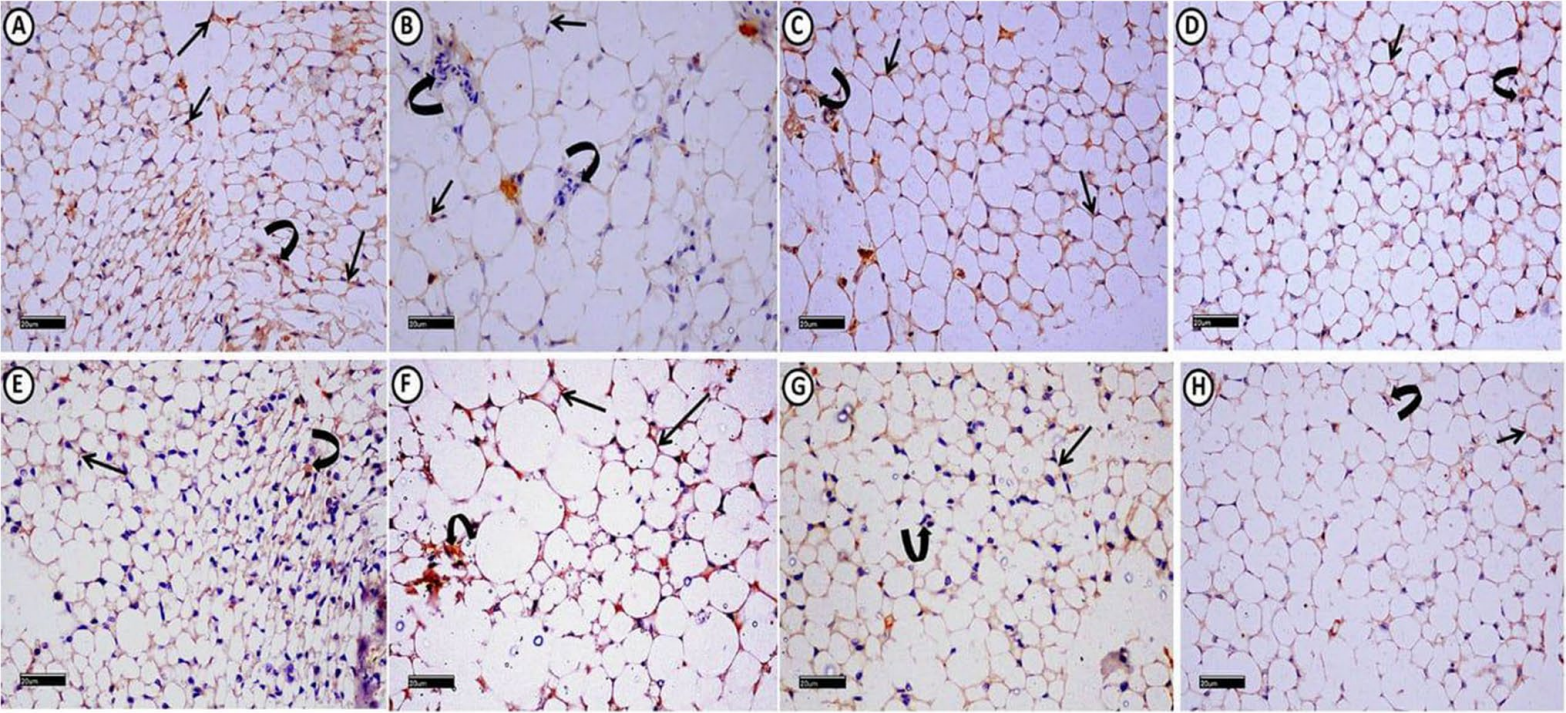
A photomicrograph of epididymal white adipose connective tissue sections from all experimental groups immuno-histochemicaly stained with: (I): IGF-1: Control group (**A**), shows extensive positive cytoplasmic immuno-reaction in most of adipocytes (arrows) and connective tissue (C.T.) cells in between adipocytes (curved arrow). MS group (**B**) shows mild immuno-reactivity in adipocytes (arrows) and negative immuno-reaction in C.T. cells (curved arrows). MS + Dulaglutide (**C**) and MS + Resveratrol (**D**) groups exhibit strong cytoplasmic immuno-reaction in both of adipocytes (arrows) and C.T. cells (curved arrows). (II): TNF-α: Control group (**E**), expresses minimal cytoplasmic immuno-reaction in adipocytes (arrow) and C.T. cells (curved arrow). In MS group (**F**), marked TNF-α immuno-reactivity is found in most of adipocytes (arrows) and C.T. cells (curved arrow). MS + Dulaglutide (**G**) and MS + Resveratrol (**H**) groups, show mild immuno-reaction in adipocytes (arrows) and C.T. cells (curved arrows) (IGF-1 immuno-stain (A, B, C, D) & TNF-α immuno-stain (E, F, G, H) Scale bar = 20 µm)

**Fig. 5:**
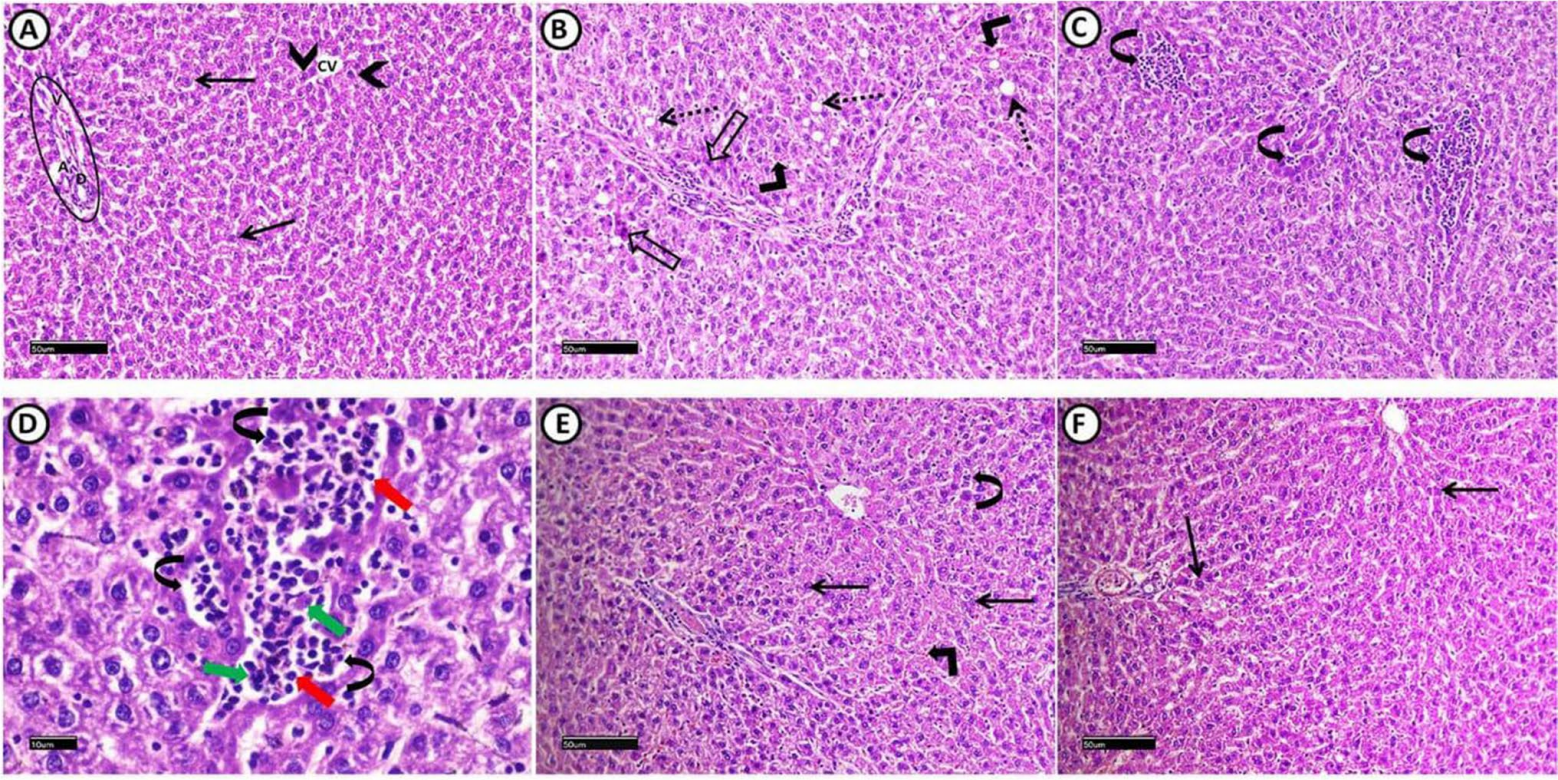
A photomicrograph of H&E-stained liver sections from all experimental groups: Control group (**A**), shows normal histological architecture with radiating cords of hepatocytes from central veins (CV). Portal area (encircled) (having branches of portal vein (V), hepatic artery (A) as well as bile duct (D)) is situated in the corners of the ill-defined classic hepatic lobule. Hepatocytes (arrows) appear with granular acidophilic cytoplasm and vesicular nuclei with prominent nucleoli, some are binucleated (arrowheads). MS group (**B**, **C**, **D**) shows many vacuolated hepatocytes with dark peripherally situated nuclei (dotted arrows). Others have deep acidophilic cytoplasm and small dark pyknotic nuclei (hollow arrows). Extravasation of erythrocytes (right-angled arrows), as well as inflammatory infiltration (curved arrows) with both of lymphocytes (red arrows) and macrophage (green arrows) are obvious findings. MS + Dulaglutide group (**E**), shows normal vesicular hepatocytes (arrows). Mild erythrocytes extravasation (right-angled arrow) and inflammatory infiltration (curved arrows) could be detected. MS + Resveratrol group (**F**) exhibits apparent normal hepatic architecture with normal hepatocytes (arrows). (H&E stain Scale bar = 50 µm (A, B, C, E, F), 10 µm (D))

**Fig. 6:**
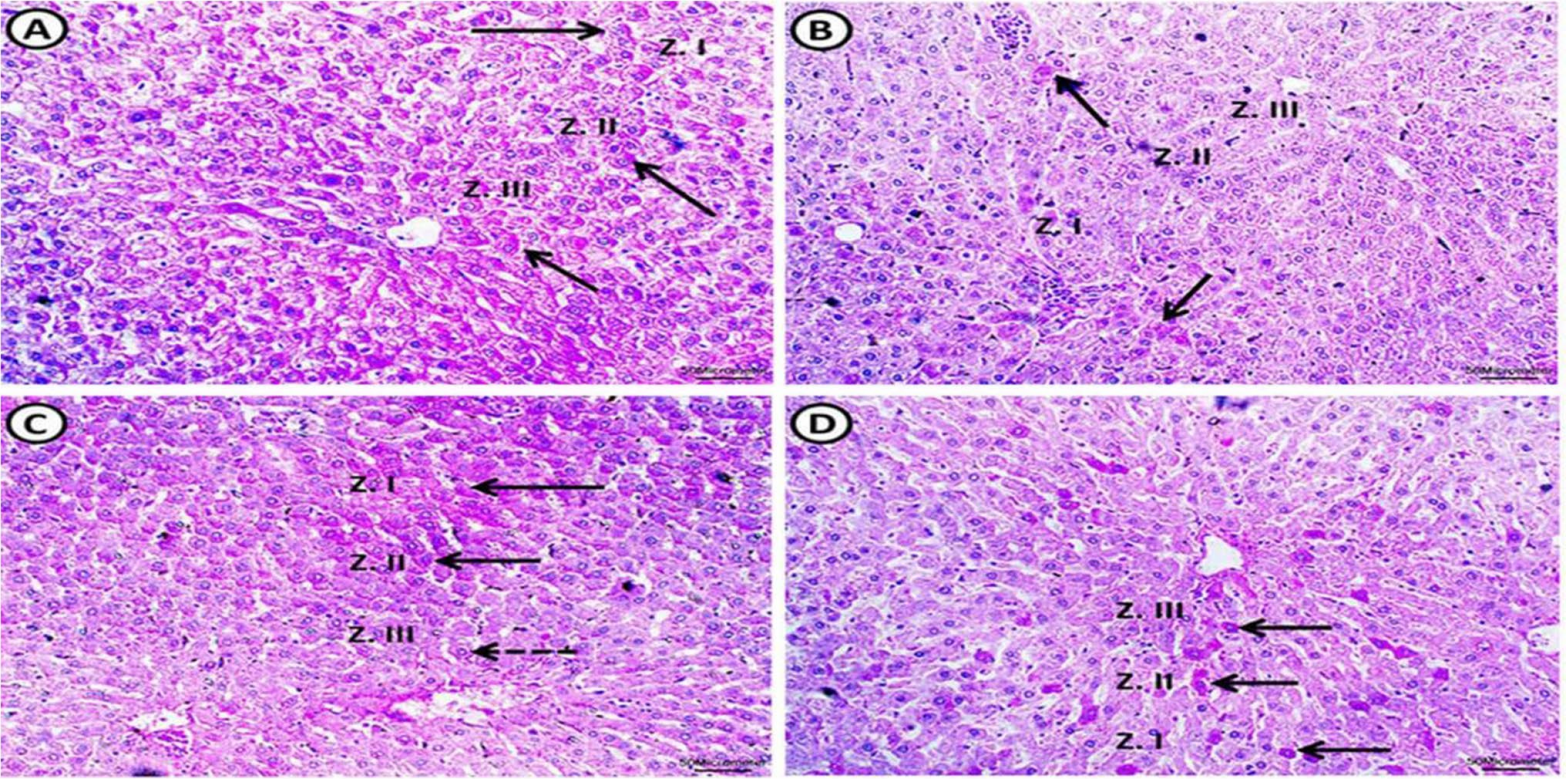
A photomicrograph of PAS-stained sections of the liver from all experimental groups: control group (**A**) shows normal distribution of glycogen in the hepatocytes. Positive histochemical reactions (arrows) are distributed mostly equal in all hepatic lobule zones: zone I (peripherally located area of classic hepatic lobule close to portal area), zone II (area between zone I and III) and zone III (area around central vein). MS group (**B**) shows apparent negative reaction in zone II and III. Few hepatocytes in zone I exhibit positive reaction for PAS (arrows). MS + Dulaglutide group (**C**) exhibits strong PAS positive reactivity (arrows) in zone I and II as well as many hepatocytes reveal moderate PAS reactivity (dotted arrow) in zone III. MS + Resveratrol group (**D**) shows intense PAS positive reactivity in sporadic cells (arrows) that are distributed apparently equal in all zones (I, II, III). (PAS stain, Scale bar = 50 µm)

**Fig. 7:**
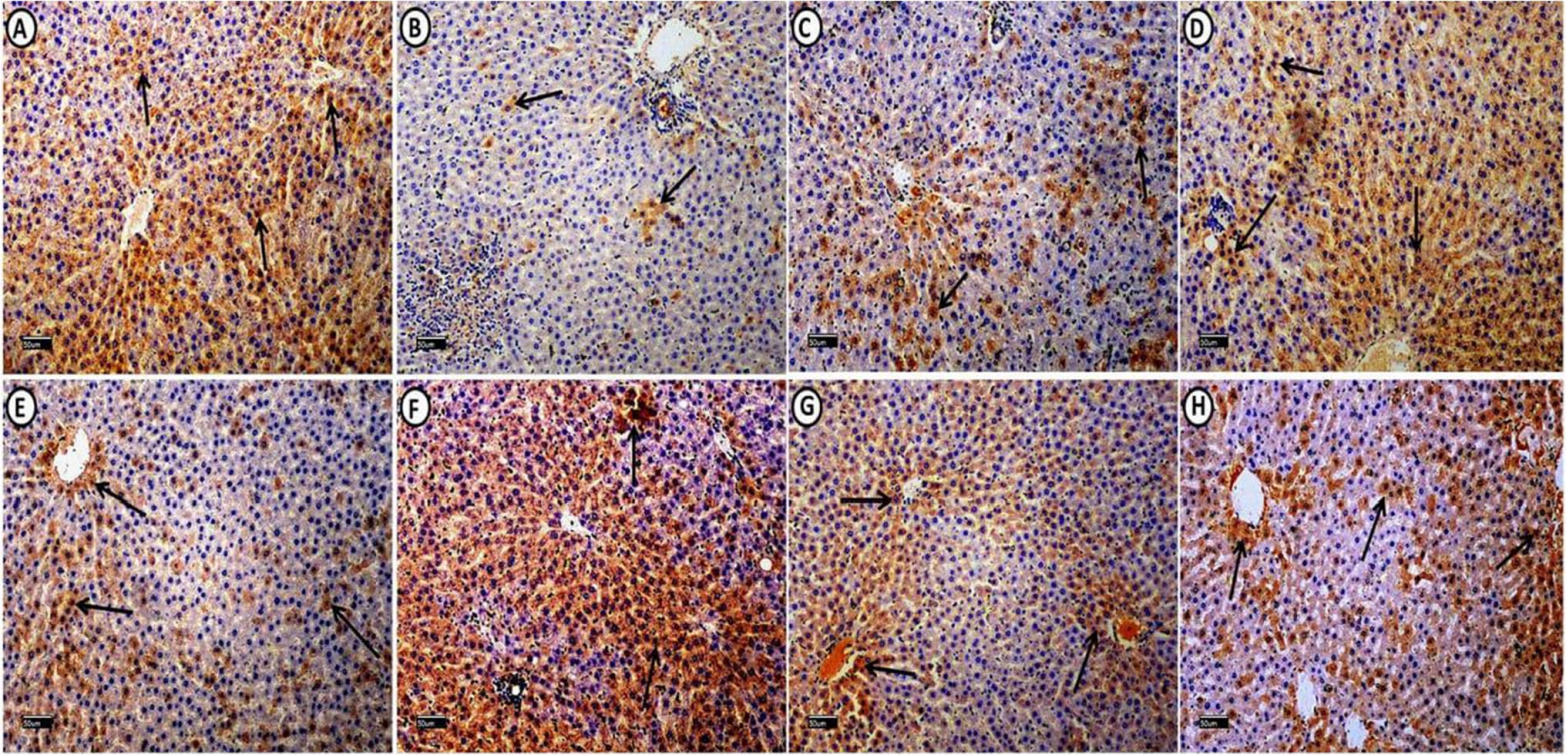
**A** photomicrograph of liver sections from all experimental groups immuno-histochemicaly stained with: **I:** IGF-1: Control group (**A**), shows extensive cytoplasmic immuno-reaction in the majority of hepatocytes (arrows). MS group (**B**) reveals that the immuno-reaction is restricted to few hepatocytes (arrows). IGF-1 immuno-staining (arrows) was detected in large number of hepatocytes in both MS + Dulaglutide (**C**) and MS + Resveratrol (**D**) groups. **II:** TNF-α: in control group (**E**), few hepatocytes express cytoplasmic immuno-reaction (arrows). MS group (**F**) showed that TNF-α immuno-stain (arrows) was found in most liver cells. In both MS + Dulaglutide (**G**) and MS + Resveratrol (**H**) groups, some hepatocytes were seen expressing the immuno-stain (arrows). (IGF-1 immuno-stain (**A**, **B**, **C**, **D**) TNF-α immuno-stain (**E**, **F**, **G**, **H**) Scale bar = 50 µm)

Examination of epididymal white adipose connective tissue (WAT) revealed average-sized adipocytes and positive cytoplasmic IGF-1 immuno-reaction in most adipocytes and connective tissue (CT) cells. TNF-α immuno-stain showed minimal cytoplasmic immuno-reaction (Figs. 3A, 4A, E). Liver examination revealed normal histological architecture with radiating hepatocytes cords from central veins. Hepatocytes had granular acidophilic cytoplasm and vesicular nuclei. Positive PAS histochemical reactions are distributed mostly equally in all zones (Figs. 5A, 6A). We detected that most cells expressed positive cytoplasmic IGF-1 immuno-reaction in contrast to TNF-α (Fig. 7A, E).

#### MS group

Adipocyte size increased significantly (*P* < 0.05) compared to the control group. Some adipocytes showed visible perivascular inflammatory cell infiltration and damaged cell borders (Fig. 3B). IGF-1 immunoreactivity in adipocytes was minimal, but immunoreactivity in CT cells was negative. Immuno-reaction area percentage decreased significantly. The area percentage of TNF-α immunoreactivity showed a significantly increase (Figs. 2B, C, 4B, F).

Upon liver inspection, we saw several vacuolated hepatocytes with dark peripheral nuclei. Other hepatocytes had deep acidophilic cytoplasm and tiny dark pyknotic nuclei. It was clear that there had been erythrocyte extravasation and inflammatory infiltration (Fig. 5B, C, D).

There was a negative reaction in zones III and II with a residual positive PAS reaction in a small number of hepatocytes in zone I. There was a much lower proportion of PAS reaction area in liver sections compared to control (Fig. 6B, Table 1). Compared to control, TNF-α reaction significantly increased, but we found IGF-1 immuno-reaction only in a small number of hepatocytes with a considerable drop in area % (Figs. 2B, C, 7F, B).

#### Dulaglutide treated group

We found large-sized adipocytes with clearly defined boundaries, but there was a considerable decrease in diameter compared to the MS group (Fig. 3C, Table 1). Compared to the MS group, there was a significant rise in IGF-1 immunoreactivity and a decrease in TNF-α (Figs. 2B, C, 4C, G). Hepatocytes appeared normal except for a small number that had black pyknotic nuclei (Fig. 5E). When compared to the MS group, there was a discernible rise in PAS reactivity (Fig. 6C, Table 1). Opposed to the MS group, there were noticeably higher levels of IGF-1 and lower levels of TNF-α immuno-expression in hepatocytes (Figs. 2B, C, 7C, G).

#### Resveratrol treated group

WAT examination revealed large-sized adipocytes with well-defined boundaries. Compared to the MS group, we detected a significantly decreased adipocyte diameter (Fig. 3D, Table 1). Significant increased IGF-1 while decreased TNF-α immune-reactivity were noticeable (Figs. 2B, C, 4D, H). Resveratrol improved the liver tissues, appearing with normal architecture (Fig. 5F). PAS reaction (area percentage) revealed a significant increase compared to the MS group. Intense PAS-positive reactivity in sporadic cells is distributed equally in all zones of the hepatic lobule (Fig. 6D, Table 1). We observed significantly increased IGF-1 immuno-reaction and decreased TNF-α compared to the MS group (Figs. 2B, C, 7D, H)**.** Resveratrol revealed a significant difference in the ameliorating effects compared to dulaglutide regarding the adipocytes diameter, the area percentage of IGF-1 in adipose and liver tissues, and TNF-α in adipose tissue. Whereas there was a non-significant difference in the area percentage of PAS reaction and TNF-α in liver tissue **(**Fig. 2B, C, Table1).

## Discussion

Chronic consumption of an unhealthy diet and a sedentary lifestyle induces metabolic complications and fatty liver [29]. In the present study, feeding rats HF/HS diet for eight weeks produced features of MS. These features included obesity markers, hyperglycemia, hyperinsulinemia, IR, dyslipidemia, increased SBP and DBP, and decreased HR. The levels of ALT and MDA were also elevated in MS rats, indicating liver dysfunction with hepatocyte damage and enhanced OS in the liver and fat tissues. However, treatment with dulaglutide or resveratrol significantly improved all these impairments. Earlier studies reported that a high-fat diet (HFD) caused glucose intolerance and enhanced tissue lipid oxidation leading to insulin resistance. Here, the observed hyperglycemia induced by HF/HS diet results in compensatory hyperinsulinemia and downregulation of glucose transporter4 expression, a mechanism attributed to IR induction [30].

In the current study, dulaglutide, a long-acting GLP-1 agonist, exhibited potential therapeutic benefits for MS-induced T2DM and obesity by direct insulin-dependent effects that improve hyperglycemia and IR. In addition, it has extra-pancreatic activities as it enhances liver and adipose glucose metabolism and reduces hepatic glycogenolysis [29]. Furthermore, dulaglutide improved all obesity indices (BW, % of BW gain, Lee index, and BMI) by decreasing appetite and delaying gastric emptying [31]. Following our findings, a previous study demonstrated a significant reduction of BW, BMI, and waist circumference (WC) in patients who used liraglutide and dulaglutide versus the conventional treatment [32]. Another study revealed that combined dulaglutide and metformin resulted in higher significant weight loss in patients with T2DM [33].

Similarly, several studies on animals and humans reported that resveratrol controls glycemic disorders in T2DM through improvement not only in insulin sensitivity but also in insulin secretion [34]. Resveratrol significantly reduced the visceral adipose tissue mass, indicating more adipokines production. Hou and his colleagues suggested that the lower fat mass and improvement of obesity markers induced by resveratrol may be through modification in the molecular levels of adipose tissue metabolism [35]. In animal and human isolated adipocytes, resveratrol decreases triglycerides content and increases lipolysis by stimulation of adenosine triglyceride-dependent lipase and adenosine monophosphate monophosphate-activated protein kinase (AMPK) [36]. In a study on HFD-fed rats, the drug also decreases fat mass by inhibiting free fatty acid synthase and acetyl CoA carboxylase in different depots. In agreement with our findings, resveratrol significantly decreased BW, fat mass, WC, BMI, and insulin secretion in patients with MS in a randomized, double-blind, placebo-controlled clinical trial [37].

The histological analysis of adipose tissue in the MS group indicated increased adipocyte size (i.e. hypertrophy), perivascular cell infiltration and increased TNF-α reactivity. This hypertrophy may result in adipocyte necrosis that promotes macrophage infiltration, the main source of the pro-inflammatory cytokine TNF-α [38]. Furthermore, the hypertrophied adipocytes express less IGF-1, evoking macrophages recruitment which maintains IGF-1 in fat [39]. We could not detect IGF-1 expression in CT cells in the MS group; this may be due to the overwhelming oxidative and inflammatory stress that burden both adipocytes and CT cells.

IGF-1 is a crucial hormone in the pathophysiology of metabolic syndrome as it affects carbohydrate and lipid metabolism. It regulates adipocyte metabolism and suppresses lipolysis in a manner analogous to insulin [40]. In obesity, multiple adipokines such as TNF-α regulate adipocyte homeostasis, disturb the IGF-1 synthesis, and impair its signaling. These effects result in the blockade of IGF-1 benefits and indicate a link between IGF-1 and MS [41].

Also in our result, the chronic inflammation in adipose tissues and liver associated with increased TNF-α, a suppressor of adiponectin expression may decrease the adiponectin levels in MS group. This in agreement with previous researchers who suggested that the reduced adiponectin concentration in obese patients with increased adipose mass may be due to increase in TNF-α expression [42].

One of the beneficial effect of adiponectin on MS may be through PPARγ [43]. Our results showed a strong positive correlation between adiponectin and PPARγ levels in liver and adipose tissues. PPARγ is an essential regulator of adipogenesis and necessary for pre-adipocyte differentiation [43]. A previous study reported that PPARγ agonist decreased visceral fat and steatohepatitis disease activity [44]. In the present study, the expression of hepatic and adipose PPARγ is significantly down-regulated in MS rats, while treatment with resveratrol and dulaglutide significantly up-regulated it.

Several studies demonstrated reduced adiponectin levels in obesity, IR, and MS. Adiponectin, an adipocyte-derived hormone with anti-inflammatory properties, affects energy homeostasis, glucose, and lipid metabolism [7, 42, 45]. In the present study, we can suggest that the significantly decreased adiponectin level in the MS group may deteriorate the insulin sensitivity, lipid profile, obesity markers, and TNF-α reactivity.

Furthermore, resveratrol and dulaglutide notably alleviate these MS components, and tissue inflammation by increasing tissue IGF-1, PPARγ, and adiponectin levels, decreasing TNF-α, consequently improving IR, adiposity, and lipid profile. Previous work revealed that resveratrol improved glucose and insulin tolerance due to reduced serum glucose and insulin, and increased adiponectin in KKA(y) mice [46]. In adipose tissues, resveratrol also increases the production of adiponectin and peroxisome proliferator-activated receptor coactivator-1(PGC-1α), resulting in increased mitochondrial biogenesis and energy expenditure [15].

Histological examination in the MS group also revealed marked hepatic injury, indicating risks for fatty liver. These include a significant amount of liver cell degeneration and inflammatory infiltration alongside an abundance of vacuolated cells, most of which may be fat deposits dissolved during tissue preparation. Increased lipolysis from adipose tissue is the primary cause of fat accumulation in the liver [47].

Additionally, the observed substantial decline in hepatic glycogen content was consistent with a previous result [48]. The difference in oxygenation and metabolic activity in these zones may be due to the variation in hepatic lobule zone affection [49]. Glycogen depletion in MS may result from the liver's attempt to counteract the stress brought on by high energy intake [50], or it may be due to an alteration in its synthesis produced by hepatocyte degeneration. In addition, the widespread inflammation and oxidative stress that assault hepatocytes may cause decreased hepatic IGF-1 immuno-reactivity and elevated MDA and serum ALT levels in MS. In this issue, Völzke et al. declared an association between hepatic steatosis and low serum IGF-1 levels [51].

Accordingly, in a study on the murine NASH model, GLP-1 agonists improved BW, glycemic profile, hepatic enzymes, and histology as well as OS by increasing fatty acid oxidation and decreasing hepatic lipogenesis [52]. In patients with T2DM, dulaglutide treatment reduced BW and glycemic profile resulting in significant reduction of serum liver enzymes [53]. In agreement with our findings, Gameil et al. found that liraglutide and dulaglutide treatment in diabetic patients exhibited a significant improvement of HBA1c, lipid profile, SBP, and DBP in diabetic patients [32].

In the same context, some mechanisms to control glycemic homeostasis by resveratrol may be due to increasing insulin signaling pathway activity in rat liver [54]. Besides, it possibly via regulating genes involved in glucose metabolism favoring reduced hepatic glucose synthesis and efflux [55]. Moreover, resveratrol reduces fat mass through promoting fatty acid oxidation in liver and skeletal muscle, enhancing energy disbursement and possibly increasing lipolysis [13]. Resveratrol also modulates blood pressure through several mechanisms, including AMPK phosphorylation, elevated NO levels, SIRT1 activation, and reduced production of reactive oxygen species by regulating SOD2, NADP oxidase, and glutathione reductase [56].

Furthermore, our results observed increased leptin concentration in adipose and liver tissues in the MS group that was corrected after dulaglutide and resveratrol treatment. A prior study reported that leptin regulates energy homeostasis by decreasing appetite [57]. In our work, MS-induced obesity may lead to leptin signaling dysfunction, consequently, leptin resistance to food intake and body weight control [58].

The current work showed a negative correlation between leptin and adiponectin. Generally, the effect of leptin on adiponectin differs according to the degree of adipocyte differentiation. Leptin stimulates adiponectin expression in differentiated white pre-adipocytes, an action that deteriorates in obese people. Here, MS-induced obesity may help to explain this disparity [6].

Leptin is a pro-inflammatory cytokine that elevates inflammatory markers like TNF-α [59]. This supports our findings of the direct correlation between leptin and TNF-α levels and the inverse association with adiponectin. One possible mechanism through which dulaglutide and resveratrol lowered liver and adipose tissue inflammation was through their leptin-decreasing impact. Treatment with resveratrol can result in hypoleptinemia and appears to lessen leptin resistance [21]. According to our data, no study reported an association between dulaglutide and tissue leptin levels.

Furthermore, SIRT-1 is the primary modulator for the hepatic metabolism of lipids and glucose [60]. The decreased hepatic SIRT-1 expression after MS induction is consistent with the previous researchers who reported that the pathophysiology in CVS, T2DM, liver steatosis, and MS in mice with high fatty acid enriched diet is due to reduced SIRT-1 expression and activity [61]. Because SIRT-1 increases during weight loss and decreases in obese patients, the current findings may imply that SIRT-1 reduction plays a crucial role in MS disorders such as T2DM, IR, obesity, and NAFLD [62].

Several mechanisms of SIRT-1 are beneficial to alleviate these disorders, including the decreased risk of liver steatosis in response to a high-fat diet via modifying lipogenesis and liver fat export [63]. Other research reported that SIRT-1 inhibitors decreased adiponectin and PPARγ production in adipocytes. The study elucidated a positive impact of SIRT-1 to up-regulate adiponectin expression [64]. Also, SIRT-1 deprived liver exhibited a reduction of PPAR γ transcription target fatty acid oxidation genes [65].The above reports could explain our observation of the direct correlation among SIRT-1, PPARγ, and adiponectin. Furthermore, hypothalamic SIRT-1 over-expression can control food intake and BW gain through increased leptin sensitivity in obesity [66]. An earlier study showed that SIRT-1 controls cholesterol homeostasis by regulating the transcription of the liver X receptors (LXRs) target genes [67].

The abovementioned mechanisms may partly explain our findings in improved MS-induced obesity and liver dysfunction after dulaglutide or resveratrol treatment via increased hepatic SIRT-1 expression. Previous authors reported that the SIRT-1 /autophagy pathway is crucial as the protective effects of resveratrol against HFD -induced hepatic steatosis [68]. Similarly, an earlier study revealed that dulaglutide increased SIRT-1 in endothelial tissues, but little data is available on its role on hepatic SIRT-1 in NAFLD [69].

The substantial negative correlation between SIRT-1, TNF-α, and leptin suggests that SIRT-1 plays a role in mediating the anti-inflammatory effects of resveratrol and dulaglutide. It may be through the regulation of NF- κB transcriptional activity, which lowers TNF-α expression [70].

Several studies reported other mechanisms of the anti-inflammatory effects of dulaglutide and resveratrol. Previous animal and human studies suggested that the gut microbiome contributes to the pathogenesis of NASH, and microbiome dysbiosis increases the flux of microbial endotoxins to the liver, further enhancing the pro-inflammatory and pro-fibrotic processes [71]. In a study on mice with a high-fat-high-carbohydrate diet, dulaglutide improved MS and induced hepatic anti-inflammatory effects by improving microbiome dysbiosis [72]. Other study showed that resveratrol modulates gut microbiota and their metabolic derivatives, such as intraluminal lipids and short-chain fatty acids (SCFAs), hence alleviating metabolic syndrome [73].

Finally, we can suggest that the possible mechanisms of resveratrol and dulaglutide to improve HF/HS diet-induced MS in the rat model may partly be due to alleviating hyperglycemia, hyperinsulinemia, IR, dyslipidemia, and hypertension. Alongside, anti-inflammatory and antioxidant effects- induced by these compounds participate in modulating the function and histology of liver and adipose tissues, mainly by modulating the cytokines and adipokines levels and activities, particularly SIRT-1, adiponectin, leptin, IGF-1, and PPARγ.

The current study has some limitations; we recommend further studies to evaluate other mechanisms by which resveratrol and dulaglutide affect MS features, such as their effects on the microbiome, thromboembolic complications, and chronic kidney disease. In addition, we did not assign a model group with combined resveratrol and dulaglutide therapy to compare the potential the potential synergistic and separate effects.

### Conclusion

The present study revealed that both resveratrol and dulaglutide improved MS disorders. Particularly the reduction of IR, obesity, fatty liver, chronic low-grade inflammation, and OS. The drugs induced their beneficial effects partly through the improvement of SIRT-1 expression that may be responsible for the increased adiponectin, PPARγ, and IGF-1, and decreased leptin, TNF-α, and MDA levels. Consequently, promoting the integrity and function of hepatic and adipose tissues occurs. Moreover, resveratrol significantly affects the hemodynamics, lipids, adipokines (adiponectin, leptin, TNF-α), and IGF-1 levels more than dulaglutide does, while dulaglutide has more influence on glycemic control. Our study declared several therapeutic benefits of dulaglutide as well as resveratrol. So, we recommend using these drugs to treat obesity, fatty liver, and T2DM in patients with metabolic syndrome.

## Declarations

We confirm that this work is original and has not been published elsewhere (partly or in full).
